# Quantity and Variety of Fruit and Vegetable Intake with Changes in Measures of Adiposity among Community-Dwelling Chinese Older Adults

**DOI:** 10.3390/nu15194096

**Published:** 2023-09-22

**Authors:** Shu-Yi Li, Jason C. S. Leung, Zhi-Hui Lu, Timothy C. Y. Kwok

**Affiliations:** 1 Department of Medicine and Therapeutics, Prince of Wales Hospital, The Chinese University of Hong Kong, Hong Kong, China; 2 Jockey Club Centre for Osteoporosis Care and Control, The Chinese University of Hong Kong, Hong Kong, China

**Keywords:** fruit, vegetable, body weight, fat mass, older adults

## Abstract

Background: The association between fruit and vegetable intake, considering both quantity and variety, and weight change among older adults remains controversial. We aimed to investigate the association between fruit and vegetable intake, variety, and changes in adiposity measures among community-dwelling Chinese older adults. Methods: A total of 2944 participants aged ≥65 years from Hong Kong communities were included. Fruit and vegetable intake was estimated by a locally validated food frequency questionnaire. Adiposity measures (weight, body mass index (BMI), waist circumference, fat and lean mass) were assessed at baseline and the year four follow-up. Restricted cubic spline and logistic models were performed to estimate the associations between fruit and vegetable intake, variety, and changes in adiposity measures. Results: A nonlinear association between vegetable intake and weight change was found, following a J-shape curve. Increased vegetable intake was associated with less increases in weight, BMI, and fat mass among those below median intakes. However, these associations became insignificant at higher intakes (all *P*-nonlinearity < 0.05). The ORs (95% CIs) for weight gain across the quartiles of vegetable intake were as follows: 1.00 (reference), 0.81 (0.56, 1.17), 0.55 (0.36, 0.83), and 0.88 (0.58, 1.33). Similar patterns were observed in overweight and normal weight participants, but not in those with low body weight. No associations with weight change were found for fruit intake or fruit and vegetable variety. Conclusions: Moderate vegetable intake was associated with less weight gain among community-dwelling Chinese older adults, but not in those with low body weight. No association was observed between fruit intake or variety with weight change.

## 1. Introduction

Global population aging is accelerating. Older adults may experience weight change due to unhealthy lifestyle factors, such as poor diet and increased sedentary behavior [1], along with age-related muscle loss, decreased appetite, and reduced energy intake [2]. Overweight and obesity are associated with an increased risk of chronic diseases, particularly cardiovascular disease [3]. Weight loss is linked to the development of sarcopenia and frailty, resulting in adverse health outcomes in older adults [4]. Recently, a meta-analysis of observational studies found that weight change (weight loss, gain, or fluctuation) was associated with an increased risk of all-cause mortality in community-dwelling adults aged ≥ 65 years, compared with weight-stable adults [5].

Lifestyle is a modifiable factor that affects body weight. A healthy dietary plan can help older adults achieve an appropriate weight, preserve lean mass, and stabilize body fat [6]. Fruits and vegetables are low-energy-density foods with high contents of dietary fiber. Dietary fiber can delay gastric emptying, increase satiety, regulate glucose metabolism, and promote gut health, which is beneficial for weight management [7]. Fruits and vegetables are also good sources of minerals (e.g., potassium and magnesium), vitamins (e.g., vitamin C, carotene, and folates), and phytochemicals (e.g., carotenoids, flavonoids, anthocyanins, and organosulfurs) [8,9]. These bioactive components in fruits and vegetables play a role in weight loss by suppressing adipose tissue growth, stimulating lipolysis, inducing apoptosis, and reducing obesity-mediated chronic inflammation and oxidation, resulting in reduced weight and adipose tissue [9,10]. However, fruits contain abundant fructose, and current research expressed concerns that fructose may promote energy storage instead of utilization, potentially contributing to obesity [11]. Inconsistent findings emerge from meta-analyses regarding the influence of increased fruit and vegetable intake on weight loss or maintaining a healthy weight [12,13,14]. Higher fruit and vegetable intake was inversely associated with weight change in prospective cohort studies conducted among United States adults [15] and Chinese adults [16]. However, no such association was observed in European adults [17]. In contrast, increased fruit intake was positively associated with weight change among Japanese middle-aged and older adults [18]. Additionally, the effects of fruits and vegetables may be different among the underweight, normal weight, and overweight. The inverse association between fruit and vegetable intake and weight change was mainly obtained among overweight participants [19], while research on individuals with low body weight was scarce [12,13,14]. Therefore, further research is required to investigate the association between fruit and vegetable intake and weight change stratified by baseline body mass index (BMI).

Previous studies have mainly focused on the association between the quantity of fruit and vegetable intake and weight change, with relatively little exploration of their variety [8]. The content of dietary fiber and bioactive compounds varies among different kinds of vegetables and fruits. Certain fruits and vegetables are rich sources of vitamin C, such as citrus fruits and dark green and leafy vegetables [20]. Yellow, orange, and red fruits and vegetables are rich in carotene and carotenoids, while allium vegetables, such as onions, contain high levels of organosulfur compounds. Anthocyanins are abundant in red and purple fruits and vegetables [10]. Greater variety in fruit and vegetable intake is associated with higher consumption of fiber, vitamins, minerals, and phytochemicals. In a previous longitudinal cohort study conducted among Tehran adults, higher intakes of red and purple fruits and vegetables were associated with reduced weight and abdominal fat gain [21]. Similarly, red or yellow vegetables and allium vegetables were inversely associated with weight gain in Japanese middle-aged and older adults, while other types of vegetables did not show the same association [18]. To our knowledge, only one longitudinal study reported the association between fruit and vegetable variety score and adiposity among older adults. This study found that a higher fruit and vegetable variety score was associated with decreases in body weight and waist circumference among the elderly Mediterranean population [8]. It is worth noting that older adults often have monotonous food choices due to their health status and economic considerations [22], but few studies have specifically targeted this age group or stratified their findings by age.

Therefore, this study aimed to examine the association between the quantity and variety of fruit and vegetable intake and 4-year changes in adiposity measures among community-dwelling Chinese older adults in Hong Kong. We also investigated whether the association between fruit and vegetable intake and weight change differs across baseline BMI.

## 2. Materials and Methods

### 2.1. Study Participants

Data were from the Mr. OS and Ms. OS (Hong Kong) cohort, a community-based prospective cohort study, which was designed to examine the determinants and risk factors of osteoporosis, sarcopenia, and other health outcomes. The details of the study protocol have been published previously [23]. In brief, 2000 Chinese men and 2000 Chinese women were recruited from local communities through advertisements and health talks between August 2001 and March 2003. A stratified sample was used to reach approximately 33% in each age group: 65–69, 70–74, and 75 years and above. Written informed consent was obtained from all participants. The study was approved by the Clinical Research Ethics Committee of The Chinese University of Hong Kong.

We excluded participants if they met one of the following exclusion criteria: (1) lack of dietary data (*n* = 5); (2) extreme energy intake (>4000 kcal/d or <800 kcal/d for men; >3500 kcal/d or <500 kcal/d for women) (*n* = 13); (3) missing data on demography or measures of adiposity at baseline (*n* = 284); (4) without any follow-up data regarding measures of adiposity (*n* = 754). Finally, 2944 participants were included in the data analysis.

### 2.2. Fruit and Vegetable Intake Assessment

Dietary intakes of fruits and vegetables were assessed at baseline by the validated 280-item food frequency questionnaire (FFQ) through face-to-face interviews by trained research staff [24]. Participants were asked about the frequency and the usual amount of food consumption of each item during the previous 12 months. A catalog of pictures of individual food portions was used to explain one serving or portion size to participants. There were seven categories in the FFQ: grains, vegetables/fruits, meats/fish/eggs, dairy products/beverages, dim sum/snacks, soups, and oil/salt/sauces.

To assess total fruit intake, the calculation considered fresh fruit and dried fruit, while excluding sugar-added canned fruits, fruit cocktails, and fruit juice due to their high added sugar content. A total of 25 fruit items were included in the data analysis. Fruits were grouped into citrus fruits, berries and drupes, pomes, tropical fruits, and melons (Table S1) [10,20,25,26,27]. To assess total vegetable intake, starchy vegetables (e.g., potato, sweet corn, lotus root, pumpkin, and taro) and preserved vegetables were excluded due to their different starch and sodium content. Vegetables were grouped into cruciferous vegetables, green vegetables, yellow/red vegetables, allium vegetables, beans and peas, and other vegetables, with a total of 46 vegetable items considered (Table S1) [10,20,25,26,27].

### 2.3. Fruit and Vegetable Variety Assessment

The details of the assessment of fruit and vegetable variety score have been reported previously [28]. Briefly, if a specific type of fruit or vegetable was consumed at least a few times per year, one point would be given for the corresponding variety score, regardless of the amount consumed. The range of possible variety scores was from 0 to 25 for fruits and from 0 to 46 for vegetables. The top three commonly consumed fruits were oranges, bananas, and apples among our study population. In terms of vegetables, choy sum, hairy melon, and lettuce were the most frequently consumed.

### 2.4. Changes in Measures of Adiposity between Baseline and the 4th Year Follow-Up

All measurements of adiposity were performed by trained research assistants using the same method and equipment at baseline and the 4th year follow-up visits. Body weight was measured using the Physician Beam Balance Scale (Healthometer, McCook, IL, USA) to the nearest 0.1 kg. Height was measured using a Holtain Harpenden stadiometer (Holtain Ltd., Crosswell, UK) to the nearest 0.1 cm. When weight and height were measured, participants took off their shoes and wore light clothing. BMI was calculated as weight in kg divided by height in m^2^. Waist circumference was measured using a measuring tape with an accuracy of 0.1 cm, when participants stood erect. Body composition was assessed by dual-energy X-ray absorptiometry. Total fat mass, total lean mass, and their percentage were automatically analyzed using a Hologic QDR 4500 W device (Waltham, MA, USA). A Hologic body composition step phantom was used to perform calibration daily. The coefficients of variation were 1.47% for fat mass and 0.84% for lean mass. Adiposity measurements were conducted twice, and the average of the readings was calculated for analysis. Changes in measures of adiposity (weight, BMI, waist circumference, fat and lean mass) were calculated as the values in the 4th year follow-up minus values at baseline.

In this study, weight change was defined as an increase or decrease of more than 5% in weight over a 4-year follow-up period. Weight gain was identified when there was a rise in weight more than 5%, while weight loss was identified when there was a reduction in weight more than 5%.

### 2.5. Covariate Assessments

Trained research staff conducted the face-to-face interviews at baseline to collect data on demographic characteristics (e.g., age, sex, education level, subjective social status), lifestyle (current smoking, alcohol drinking, physical activity), chronic disease history, and medication use of participants. Subjective social status was assessed using a 10-rung self-anchoring scale. The lowest rung represents the most undesirable; the highest rung represents the most desirable state for their standing in the community or Hong Kong. Physical activity was estimated by applying the Physical Activity Scale of the Elderly (PASE) adapted for the Chinese population in Hong Kong [29]. The calculation of chronic diseases included several common chronic conditions: diabetes, stroke, cardiovascular disease (CVD), chronic obstructive pulmonary disease (COPD), and cancer, and was divided into three groups: 0, 1, ≥2 chronic diseases.

As mentioned before, dietary intake was assessed by FFQ at baseline. To estimate the amounts of cooking oil and condiments, we took into account the standardized portion size of individual food items and the cooking methods observed among our study population. Nutrient intake (e.g., energy, fiber, vitamin C) was calculated by multiplying food intake by the nutrient content of the specified portion size according to the Chinese Food Composition Table [30] and McCance and Widdowson [31]. Diet quality index-International (DQI-I) was calculated to assess the dietary quality in a Chinese population, and the details of the calculation method have been published previously [32].

### 2.6. Statistical Analyses

Fruit and vegetable intake was categorized into four groups based on sex-specific quartiles. In men, the mean intakes of each quartile were 97.7, 187.5, 274.6, and 491.8 g/d for fruit intake, and 97.2, 169.2, 243.2, and 436.7 g/d for vegetable intake. In women, the median intakes of each quartile were 88.5, 178.0, 249.8, and 447.0 g/d for fruit intake, and 103.0, 169.5, 232.7, and 403.6 g/d for vegetable intake. To equally divide participants into four groups, quartiles of variety score were 0–6 (Q1), 7–10 (Q2), 11–14 (Q3), >14 (Q4) for fruit; 0–20 (Q1), 21–25 (Q2), 26–30 (Q3), >30 (Q4) for vegetable. Differences in baseline characteristics were compared across quartiles of fruit and vegetable intake and variety by using *t*-test or Mann–Whitney *U* test and Chi-Square test. Differences in baseline characteristics were compared across quartiles of fruit and vegetable intake and variety by using ANOVA or Kruskal–Wallis test and Chi-Square test. The 4-year changes in measures of adiposity were assessed using paired *t*-test. Mean ± standard deviation (SD) or median (interquartile range, IQR) for quantitative variables and percentages for categorical variables were presented.

Multivariate linear regression models and restricted cubic spline (RCS) regression models were performed to estimate linear and nonlinear associations of the intake and variety of fruits and vegetables with changes in measures of adiposity. There were three points located at the 10th, 50th, and 90th percentiles of intake or variety of fruits and vegetables, and the median was used as reference in the RCS models. All models were adjusted for age, sex, education level, smoking status, alcohol drinking, subjective social status (community ladder and Hong Kong ladder), physical activity and number of chronic diseases, energy intake, DQI-I, and corresponding measure of adiposity at baseline. Additionally, variety was adjusted for in the models of fruit and vegetable intake, while intake was adjusted for in the models of fruit and vegetable variety.

Multivariate logistic regression models were used to examine the associations between the intake and variety of fruits and vegetables and weight change. Odds ratios (ORs) and corresponding 95% confidence intervals (95% CIs) were presented. Model 1 was adjusted for age and sex. Model 2 was Model 1 plus adjustments for education level, smoking status, alcohol drinking, subjective social status (community ladder and Hong Kong ladder), physical activity and number of chronic diseases, energy intake, DQI-I, and corresponding measure of adiposity at baseline. Model 3 was adjusted for Model 2 plus variety in the models of fruit and vegetable intake or adjusted for intake in the models of fruit and vegetable variety. The test for a linear trend was performed by treating the median values of fruit and vegetable intake or variety in quartiles as continuous values in multivariate logistic regression models. To test the stability of results and reduce residual confounding, sensitivity analyses were also conducted by additionally adjusting for cooking oil intake, condiment intake, and red meat and processed meat intake, or excluding those not within the mean ± 3SD of fruit and vegetable intake.

To examine the association between vegetable and fruit intake, in both quantity and variety, and weight change among participants in different baseline weight statuses, participants were grouped based on baseline BMI. We defined BMI less than 20.0 kg/m^2^ as low body weight (*n* = 332) [33]. Normal weight was defined as a BMI between 20.0 and 24.9 kg/m^2^ (*n* = 1647), and overweight as a BMI greater than 25 kg/m^2^ (*n* = 965). Then, the multivariate logistic regression models were repeated in stratified analyses.

All statistical analyses were performed by using STATA (StataCorp. 2017. Stata Statistical Software: Release 15. College Station, TX, USA: StataCorp LLC). A 2-sided *p*-value < 0.05 was considered to be statistically significant.

## 3. Results

### 3.1. Baseline Characteristics

Table 1 details the study population’s baseline characteristics. The mean age of participants at baseline was 71.8 (SD 4.8) years, and 52.0% of them were men. The mean intakes were 252.5 (SD 185.4) g/d for fruits and 232.2 (SD 150.0) g/d for vegetables. The median variety scores were 10 (7–13) for fruits and 24 (20–29) for vegetables. Baseline characteristics for participants were included, and participants without follow-up information are shown in Table S2. Compared with participants included in this study, participants without follow-up information were more likely to be older, had lower weight and lean mass, consumed less fruits and vegetables, and had lower variety of fruits and vegetables, but they had similar BMI, waist circumference, and fat mass. The 4-year changes in weight, BMI, waist circumference, fat mass, and lean mass were −0.73 (SD 3.10) kg, −0.15 (SD 1.24) kg/m^2^, 1.76 (SD 6.96) cm, −0.01 (SD 2.13) kg, and −0.69 (SD 1.49) kg, respectively (Table S3). With advancing age, weight and BMI tended to decrease, while waist circumference and fat mass percentage tended to increase (all *p* < 0.001).

Baseline characteristics across the quartiles of fruit and vegetable intake and variety are presented in Tables S4 and S5. Participants in the highest quartile of fruit and vegetable intake and variety were less likely to be smokers, had higher education levels, higher weight and more lean mass, more physical activity and higher DQI-I score, and consumed more energy, fiber, and vitamin C (all *p* < 0.05). In addition, participants in the highest quartile of fruit and vegetable variety were more likely to be younger and men, and had less fat mass (all *p* < 0.001). There were no significant differences in other baseline characteristics regarding the quartiles of intake or variety of fruits and vegetables. Spearman’s rank correlation coefficients between the intake and variety of fruits and vegetables with intakes of other food groups were shown in Table S6. Intakes of fruits and vegetables were positively associated with other foods (all *p* < 0.05), except refined grains.

### 3.2. Associations between Fruit and Vegetable Intake and Changes in Measures of Adiposity

**Figure 1:**
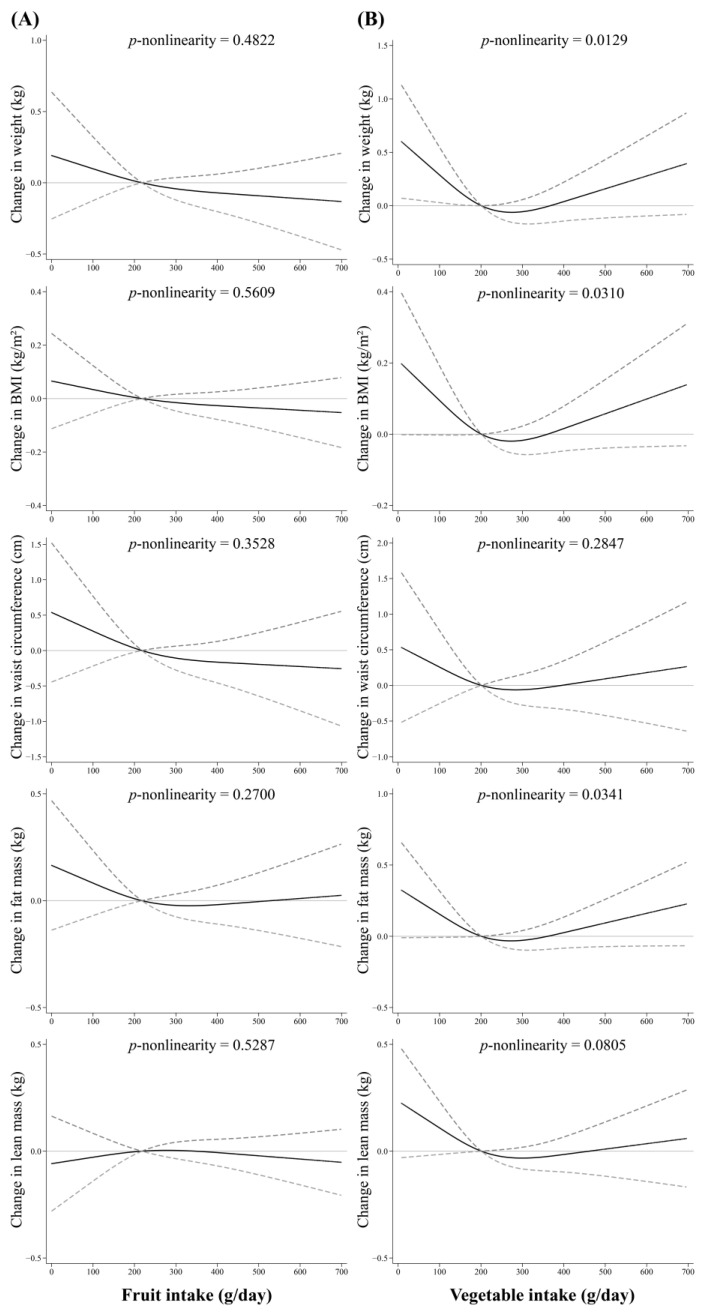
Nonlinear associations of fruit and vegetable intake with 4-year changes in measures of adiposity (weight, BMI, waist circumference, fat and lean mass). (**A**) For fruit intake; (**B**) For vegetable intake. Vegetable and fruit intake were coded using restricted cubic spline functions with three knots located at the 10th, 50th, and 90th percentiles and the median was used as reference. Adjusted for age, sex, education level, smoking status, alcohol drinking, subjective social status (community ladder and Hong Kong ladder), physical activity and number of chronic diseases, energy intake, DQI-I, baseline measures of adiposity, fruit and vegetable variety.

The associations between fruit and vegetable intake and changes in measures of adiposity are shown based on nonlinear spline models (Figure 1). Nonlinear associations between vegetable intake with 4-year changes in weight, BMI, and fat mass were found after adjusting for potential confounders, following a J-shape curve (all *P*-nonlinearity < 0.05). Increased vegetable intake was associated with less increases in weight, BMI, and fat mass among those below median intakes, while no significant association was observed at higher vegetable intake. Furthermore, yellow/red vegetables, beans, and peas showed the nonlinear association with changes in body weight and fat mass, while no association was found for other vegetables (Figures S1 and S2). However, there were no associations between intakes of total fruit and different kinds of fruits and changes in adiposity measures (Figure 1, Figures S3 and S4 and Table S7, all *p* > 0.05).

Comparable results between vegetable intake and weight change were found from the multivariate logistic regression models (Table 2). The ORs (95%CIs) for weight gain across the quartiles of vegetable intake were 1.00 (reference), 0.81 (0.56, 1.17), 0.55 (0.36, 0.83), and 0.88 (0.58, 1.33). No association was observed between fruit and vegetable intake and weight loss (Table S8). In the sensitivity analysis, there were no substantial differences after additionally adjusting for cooking oil intake, condiment intake, red and processed meat intake, or excluding those not within the mean ± 3SD of fruit and vegetable intake (Table S9).

### 3.3. Associations between Fruit and Vegetable Variety and Changes in Measures of Adiposity

**Figure 2:**
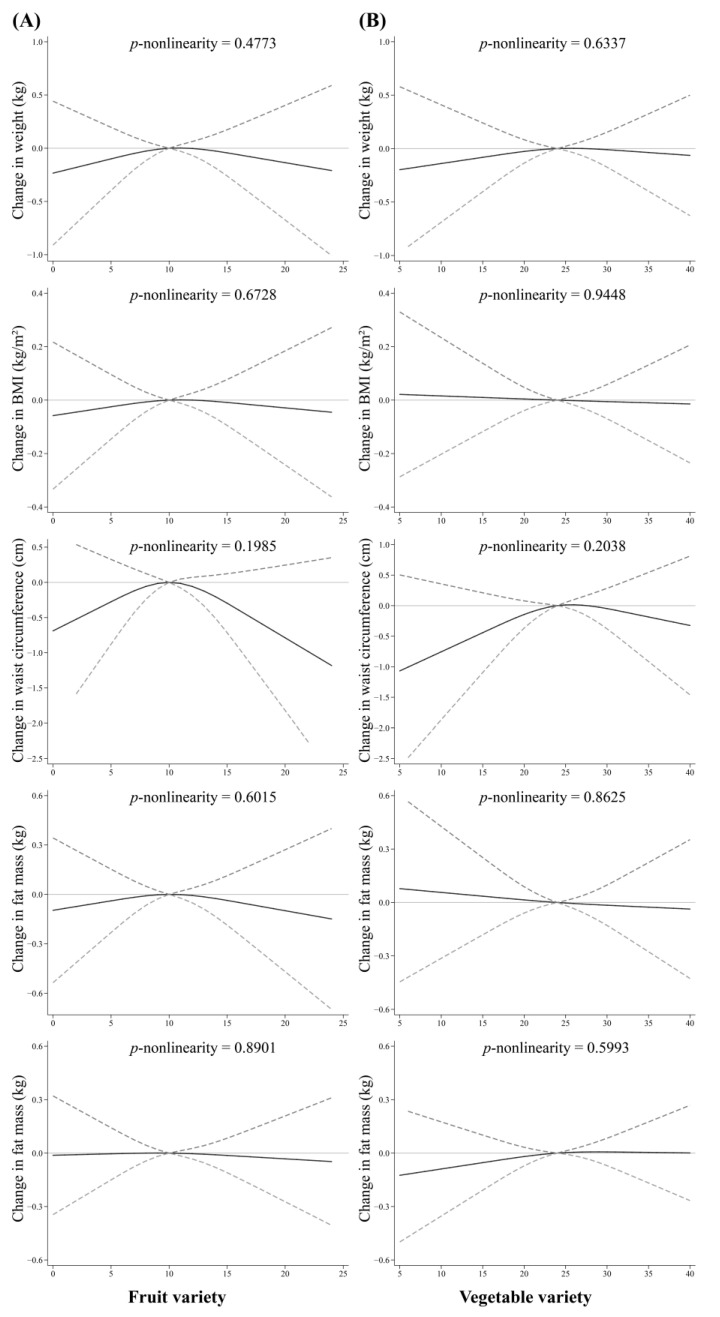
Nonlinear associations of fruit and vegetable variety with 4-year changes in measures of adiposity (weight, BMI, waist circumference, fat and lean mass). (**A**) For fruit variety; (**B**) For vegetable variety. Vegetable and fruit variety were coded using restricted cubic spline functions with three knots located at the 10th, 50th, and 90th percentiles and the median was used as reference. Adjusted for age, sex, education level, smoking status, alcohol drinking, subjective social status (community ladder and Hong Kong ladder), physical activity and number of chronic diseases, energy intake, DQI-I, baseline measures of adiposity, fruit and vegetable intake.

Fruit and vegetable variety were not associated with 4-year changes in measures of adiposity according to the results from the multivariate linear regression models and the RCS models (Figure 2 and Table S7, all *p* > 0.05). Moreover, there was no significant association between quartiles of fruit or vegetable variety and weight gain or weight loss in all logistic regression models (Table 2 and Table S8, all *p* > 0.05).

### 3.4. Associations between Fruit and Vegetable Intake and Variety and Weight Change in Different Baseline BMI

In the subgroup analysis defined by different baseline BMI, a nonlinear association between vegetable intake and weight gain was found in normal weight and overweight groups (Table 3). In the overweight group, the ORs (95%CIs) for weight gain were 1.00 (reference), 0.62 (0.29, 1.30), 0.29 (0.11, 0.74), and 0.87 (0.39, 1.94) from the first to the fourth quartile of vegetable intake. Similar results were observed in the normal weight group. However, there was no association in those with low body weight, with ORs (95%CI) for weight gain of 1.00 (reference), 1.18 (0.47, 2.95), 0.78 (0.28, 2.17), and 1.23 (0.43, 3.55) across the quartiles of vegetable intake. In addition, no significant associations between fruit intake or fruit and vegetable variety and weight gain were found across BMI categories.

## 4. Discussion

This prospective cohort study found a nonlinear association between vegetable intake and weight change, following a J-shape curve. Moderate vegetable intake was associated with less increase in weight and fat mass among community-dwelling Chinese older adults, especially in normal weight and overweight participants. However, consuming higher quantities of vegetables did not offer additional risk reduction for weight gain. No associations with weight change were found for fruit intake or fruit and vegetable variety.

Most fruits and vegetables are widely recognized for their low energy density, minimal dietary fat content, and abundant dietary fiber. These characteristics make them beneficial for weight control [34]. Previous epidemiological studies have generally reported that increasing fruit and vegetable intake was inversely associated with weight change [12,13,15,16]. However, a meta-analysis of cohort studies showed an inverse association between fruit intake and weight change, but not for vegetables [35], while increased fruit intake was positively associated with weight change in Japanese middle-aged and older adults [18]. Moreover, a meta-analysis study of randomized controlled trials reported no discernible effect on weight change [14]. In our study, moderate vegetable intake was associated with less increases in body weight and fat mass, which was consistent with findings from previous studies [12,13,15,16]. Nonetheless, the association became insignificant at the highest intake of vegetables. This nonlinear association in our study aligned with the nonlinear associations between vegetable intake amd all-cause and cause-specific mortality [36], and type 2 diabetes [37,38]. Benefits appear to be the maximum at a moderate intake of three servings (ranging from 240 to 300 g/d) of vegetables, while higher intake was not associated with additional risk reduction [36,37,38]. However, less than a quarter of our population (22.4%) met the recommended intake of 300 g/d of vegetables, as recommended in the Dietary Guidelines for Chinese Residents (2016) [39]. In traditional Chinese cuisine, vegetables are typically consumed as part of cooked dishes during main meals, whereas fruits are commonly eaten raw. Raw vegetables tend to provide superior health benefits compared to cooked vegetables [40]. In this study, vegetable intake was positively associated with cooking oil and condiment intake (Table S6). While vegetables consumed during main meals may replace high-energy foods, the addition of oil and condiments can stimulate appetite and lead to an overall increase in energy intake. However, even after adjusting for oil and condiment intake, or red and processed meat intake, the nonlinear association between vegetable intake and weight gain remained unchanged (Table S9). Furthermore, despite the rich fructose content in fruits [11], most people consume insufficient quantities of fruit, falling short of recommended levels [41]. The current levels of fruit intake did not pose a risk of increasing energy intake. In this study, 43.7% of participants met the recommended minimal fruit intake of 200 g/d [39]. Fruit intake in our population (252.5 g/d) was relatively higher than fruit intake levels observed in European adults (193.7 g/d) [42], Japanese middle-aged and older adults (169.8 g/d) [18], as well as the Chinese general population (50.0 g/d) [43]. Overall, the inconsistent association between fruit and vegetable intake and weight change may be attributed to variations in eating habits, culinary culture, food availability, and study population across different geographical regions [41].

Moderate vegetable intake was associated with less increase in fat mass, as was peas, but not for lean mass. In contrast, an inverse association with fat mass was found for fruit intake, but not for vegetable intake, in a cross-sectional study among Canadian adults [41]. Unlike our study, this study included starchy vegetable intake, such as French fries, which may increase the risk of obesity [44]. In our study, yellow/red vegetables, beans, and peas showed a significant association with less increase in fat mass, but this was not found for other vegetables (Figures S1 and S2). Previous studies also reported an inverse association between intake of yellow/red vegetables and weight change [18,21]. Yellow/red vegetables are rich in carotene and carotenoids, while isoflavones and flavonoids have been identified and characterized in beans and peas [25]. Age-related adipose tissue dysfunction in older adults promotes adipose tissue accumulation, oxidative stress, and inflammation [45]. These phytochemicals possess anti-inflammatory and antioxidant properties to suppress adipose tissue growth, inhibit preadipocyte differentiation, stimulate lipolysis, and induce adipocyte apoptosis, leading to a reduction in adipose tissue mass [10,25,46]. On the other hand, lean body mass is directly influenced by dietary protein intake. Fruits and vegetables, low in protein, may have a minor direct impact on lean body mass, but may indirectly influence it through their anti-inflammatory properties [47]. More studies are warranted to examine the potential mechanism between fruits and vegetables and body composition among older adults.

A nonlinear association between vegetable intake and risk of weight gain was found in normal weight and overweight groups, but no association was found for those with low body weight. These findings were consistent with the inverse association between fruit and vegetable intake and adiposity mainly observed among overweight adults and children in both experimental interventions and longitudinal studies [19]. Individuals with higher body weight are more prone to weight fluctuations and susceptible to the impacts of dietary and other lifestyle factors on weight [19]. A cross-sectional study among middle-aged Australian adults aged above 45 years found that obese adults were more likely to consume higher fruit and vegetable intake than their normal weight counterparts, while underweight adults were at risk for low intake of fruits and vegetables [48]. A reverse causality relationship may exist, whereby overweight and obese individuals are highly motivated to consume more fruits and vegetables as a proactive measure to lose weight. Furthermore, due to the small sample size of individuals with low body weight (*n* = 332), there may not be sufficient statistical power to demonstrate significant differences.

Few studies have examined the association between the variety of fruits and vegetables (measured by continuous scores or color groups) and changes in adiposity measures [8]. The null association between fruit and vegetable variety and 4-year changes in adiposity in this study aligned with the findings obtained from the longitudinal study in middle-aged participants with type 2 diabetes in the UK [49]. Additionally, long-term follow-up studies involving large European [50] and American [51] populations revealed no significant association between variety in fruits and vegetables with incident coronary heart disease and stroke. However, a prospective study found that greater variety of fruits and vegetables was associated with decreases in fasting blood glucose, body weight, and waist circumference among the elderly Mediterranean population at high cardiovascular risk [8]. The null association in this study may be attributed to the relatively narrow range and small differences observed among variety scores in each group (Table S5). Simultaneously, the variety of fruits and vegetables consumed by the elderly may be influenced by economic circumstances, social support, health conditions, cognitive status, and food accessibility [52]. The inconsistent results may be due to differences in these confounding factors. Despite the inconsistent findings, it remains incontrovertible that increasing the variety of fruits and vegetables results in a corresponding increase in consumption, while also increasing the intake of fiber, vitamins, minerals, and phytochemicals [50,53]. Furthermore, a cross-sectional analysis in an elderly Mediterranean population observed that greater variety of fruits and vegetables showed better dietary quality and more physical activity [53]. Considering that older adults need to prevent both muscle and bone loss as well as overweight and obesity simultaneously [6], more studies are needed to investigate the effect of variety or specific types of fruits and vegetables on weight change among older adults.

This study has several strengths, such as the relatively large sample size of community-dwelling older adults. Our study investigated the association between fruit and vegetable intake and changes in measures of adiposity among older adults, considering both quantity and variety, in contrast to previous research, which had primarily considered quantity rather than variety. Moreover, to examine whether the association between fruit and vegetable intake and weight change differs in baseline weight status, a subgroup analysis stratified by BMI categories was conducted. Furthermore, the data analysis encompassed a multitude of potential confounding factors, including physical activity and dietary quality index. However, some limitations should be noted. Firstly, dietary intake was estimated by using FFQ, which may induce recall bias. Secondly, intakes of fruits and vegetables were solely assessed at baseline and the changes in intakes during the study period cannot be included in the data analysis. In addition, our data were collected at baseline between 2001 and 2003, which was two decades ago. The total consumption of combined fruits and vegetables remained similar from 2005–2007 to 2018–2020 among Hong Kong adults aged 18 and above according to the Hong Kong Population-based Food Consumption Survey [54]. However, with the increasing convenience of transportation and economic development, there may be an increase in the variety of vegetables and fruits available. Although a study indicated that over half of older adults maintained stable dietary habits over a 10-year follow-up period [55], it is important to note that our participants’ dietary habits may have changed during follow-up. Therefore, caution should be taken when interpreting the results. Thirdly, the motivations for intentional weight loss or weight gain among older adults were not investigated in our study, which could have influenced the results in different ways. Fourthly, participants, who volunteered for the study had higher education levels and were more health-conscious than the general Hong Kong population. The prevalence of several common chronic diseases (diabetes, CVD, stroke, COPD, cancer) in our study was lower than that among residents aged 65 and over in Hong Kong [56]. This suggested that our study participants may have a relatively better health status. Thus, the results may not be generalizable to other populations.

## 5. Conclusions

Our study found a nonlinear association between vegetable intake and changes in measures of adiposity, following a J-shape curve. Moderate vegetable intake was associated with less weight and fat mass gain among community-dwelling Chinese older adults, but not in those with low body weight. However, consuming higher quantities of vegetables did not offer additional risk reduction for weight gain. No association was observed between fruit intake or fruit and vegetable variety and weight change. Further longitudinal studies and randomized clinical trials are needed to determine the role of fruit and vegetable intake, in terms of both quantity and variety, in influencing weight change among older adults.

## Figures and Tables

**Table 1:** Baseline characteristics of participants (*n* = 2994) ^1^.

Characteristics	Study Population
Fruit intake, g/day	252.5 ± 185.4
Vegetable intake, g/day	232.2 ± 150.0
Fruit variety	10 (7–13)
Vegetable variety	24 (20–29)
Age, year	71.8 ± 4.8
Male, %	52.0
Education, %	
No education	18.3
Primary or below	51.0
Secondary or above	30.7
Current smoker, %	6.4
Current alcohol drinker, %	14.0
Subjective social status ^2^, rung	
Community Ladder	6.9 ± 2.2
Hong Kong Ladder	4.6 ± 1.9
Number of chronic diseases ^3^, %	
0	62.1
1	27.7
≥2	10.3
PASE score	95.7 ± 44.0
Energy intake, kcal/day	1866.7 ± 577.1
DQI-I	64.8 ± 9.3
Fiber intake, g/day	9.4 ± 4.4
Vitamin C, mg/day	159.4 ± 96.9
Weight, kg	58.9 ± 9.6
BMI, kg/m^2^	23.7 ± 3.2
Waist circumference, cm	86.3 ± 9.2
Fat mass, kg	17.2 ± 5.2
Fat mass, %	29.3 ± 7.2
Lean mass, kg	41.2 ± 7.4
Lean mass, %	70.7 ± 7.2

Abbreviation: PASE, Physical Activity Scale for the Elderly; DQI-I, Diet quality index-International; BMI, body mass index. ^1^ Mean ± SD or median (IQR) and percentages (%) were presented. ^2^ 10-rung self-anchoring scale (the lowest rung represents the most undesirable; the highest rung represents the most desirable state for their standing in the community/Hong Kong). ^3^ The calculation of chronic diseases included diabetes, stroke, CVD, COPD, and cancer.

**Table 2:** Associations between the intake and variety of fruits and vegetables and weight gain (*n* = 2944) ^1^.

	*n* (%)	Weight Gain, OR (95%CI)		
		Model 1 ^2^	Model 2 ^3^	Model 3 ^4^
Fruit intake				
Q1	68 (9.3)	1.00 (Ref)	1.00 (Ref)	1.00 (Ref)
Q2	70 (9.5)	1.02 (0.72, 1.46)	1.06 (0.73, 1.53)	1.06 (0.73, 1.54)
Q3	64 (8.7)	0.95 (0.66, 1.36)	0.97 (0.66, 1.43)	1.00 (0.67, 1.48)
Q4	63 (8.6)	0.87 (0.61, 1.26)	0.89 (0.58, 1.35)	0.93 (0.61, 1.42)
*p*-trend		0.392	0.475	0.628
Vegetable intake				
Q1	76 (10.3)	1.00 (Ref)	1.00 (Ref)	1.00 (Ref)
Q2	66 (9.0)	0.85 (0.59, 1.20)	0.82 (0.57, 1.18)	0.81 (0.56, 1.17)
Q3	48 (6.5)	0.58 (0.39, 0.85)	0.56 (0.37, 0.84)	0.55 (0.36, 0.83)
Q4	75 (10.2)	0.96 (0.68, 1.36)	0.90 (0.60, 1.35)	0.88 (0.58, 1.33)
*p*-trend		0.856	0.785	0.730
Fruit variety				
Q1	68 (9.1)	1.00 (Ref)	1.00 (Ref)	1.00 (Ref)
Q2	83 (9.7)	1.09 (0.77, 1.53)	1.04 (0.74, 1.48)	1.04 (0.74, 1.48)
Q3	68 (9.3)	1.05 (0.73, 1.51)	1.03 (0.71, 1.49)	1.04 (0.71, 1.50)
Q4	46 (7.5)	0.80 (0.53, 1.19)	0.74 (0.49, 1.12)	0.75 (0.49, 1.15)
*p*-trend		0.300	0.207	0.239
Vegetable variety				
Q1	72 (8.9)	1.00 (Ref)	1.00 (Ref)	1.00 (Ref)
Q2	52 (7.6)	0.87 (0.60, 1.27)	0.86 (0.58, 1.26)	0.90 (0.61, 1.32)
Q3	84 (10.3)	1.16 (0.82, 1.62)	1.13 (0.80, 1.59)	1.19 (0.84, 1.70)
Q4	57 (9.0)	1.03 (0.71, 1.51)	0.99 (0.67, 1.47)	1.04 (0.69, 1.54)
*p*-trend		0.568	0.743	0.583

Abbreviation: OR, odd ratio; 95%CI, 95% confidence interval; Ref, reference. ^1^ “Weight gain” was defined as a 5% or greater gain of body weight. ^2^ Model 1: adjusted for age and sex. ^3^ Model 2: adjusted for Model 1 + education level, smoking status, alcohol drinking, subjective social status (community ladder and Hong Kong ladder), physical activity and number of chronic diseases, energy intake, DQI-I and baseline body weight. ^4^ Model 3: adjusted for Model 2 + fruit and vegetable variety for their intake or adjusted fruit and vegetable intake for their variety.

**Table 3:** Associations between the intake and variety of fruits and vegetables and weight gain over 4 years stratified by BMI categories at baseline (*n* = 2944) ^1^.

	BMI < 20.0 kg/m^2^ (*n* = 332)	BMI 20.0–24.9 kg/m^2^ (*n* = 1647)	BMI ≥ 25.0 kg/m^2^ (*n* = 965)
Fruit intake			
Q1	1.00 (Ref)	1.00 (Ref)	1.00 (Ref)
Q2	1.38 (0.78, 2.47)	1.12 (0.69, 1.84)	1.06 (0.50, 2.23)
Q3	0.92 (0.49, 1.75)	1.16 (0.53, 1.94)	0.79 (0.34, 1.81)
Q4	1.21 (0.62, 2.35)	0.86 (0.32, 1.53)	1.19 (0.51, 2.76)
*p*-trend	0.466	0.508	0.735
Vegetable intake			
Q1	1.00 (Ref)	1.00 (Ref)	1.00 (Ref)
Q2	1.18 (0.47, 2.95)	0.63 (0.38, 1.04)	0.62 (0.29, 1.30)
Q3	0.78 (0.28, 2.17)	0.49 (0.28, 0.85)	0.29 (0.11, 0.74)
Q4	1.23 (0.43, 3.55)	0.91 (0.53, 1.56)	0.87 (0.39, 1.94)
*p*-trend	0.789	0.786	0.968
Fruit variety			
Q1	1.00 (Ref)	1.00 (Ref)	1.00 (Ref)
Q2	1.36 (0.79, 2.33)	1.15 (0.72, 1.84)	0.81 (0.41, 1.60)
Q3	1.57 (0.89, 2.76)	1.14 (0.70, 1.85)	0.55 (0.25, 1.24)
Q4	0.93 (0.48, 1.82)	0.85 (0.48, 1.50)	0.69 (0.30, 1.51)
*p*-trend	0.324	0.654	0.215
Vegetable variety			
Q1	1.00 (Ref)	1.00 (Ref)	1.00 (Ref)
Q2	1.17 (0.51, 2.69)	1.01 (0.59, 1.73)	0.61 (0.29, 1.32)
Q3	1.67 (0.67, 4.15)	1.45 (0.90, 2.35)	0.93 (0.46, 1.88)
Q4	0.89 (0.31, 2.54)	1.19 (0.70, 2.04)	0.76 (0.33, 1.73)
*p*-trend	0.917	0.309	0.631

Abbreviation: OR, odd ratio; 95%CI, 95% confidence interval; Ref, reference. ^1^ “Weight gain” was defined as a 5% or greater gain of body weight. Odd ratios (ORs) and 95% confidence intervals (95%CIs) are presented. The logistic regression models were adjusted for age and sex, education level, smoking status, alcohol drinking, subjective social status (community ladder and Hong Kong ladder), physical activity and number of chronic diseases, energy intake, DQI-I and baseline body weight; they were additionally adjusted for fruit and vegetable variety for their intake or adjusted for fruit and vegetable intake for their variety.

## Data Availability

The data that support the findings of this study are available from the corresponding author upon reasonable request.

## Supplementary Materials

The following supporting information can be downloaded at: https://www.mdpi.com/article/10.3390/nu15194096/s1, Table S1. Different kinds of fruits and vegetables; Table S2. Baseline characteristics of participants included and participants without follow-up information; Table S3: Changes in measures of adiposity over 4 years in the Mr. OS and Ms. OS (Hong Kong) study; Table S4: Baseline characteristics of participants according to the quartiles of fruit and vegetable intake; Table S5: Baseline characteristics of participants according to the quartiles of fruit and vegetable variety; Table S6. Spearman’s rank correlation coefficient between the intake and variety of fruit and vegetable with intakes of other food groups; Figure S1. Associations of various kinds of vegetables intake with 4-year change in body weight; Figure S2. Associations of various kinds of vegetables intake with 4-year change in fat mass; Figure S3. Associations of various kinds of fruits intake with 4-year change in body weight; Figure S4. Associations of various kinds of fruits intake with 4-year change in fat mass; Table S7: Coefficient of association between the intake and variety of fruit and vegetable with changes in measures of adiposity over 4 years; Table S8: Associations between the intake and variety of fruit and vegetable with weight loss; Table S9: Sensitivity analysis of the association between the intake and variety of fruit and vegetable with weight gain over 4 years.
