# Supplementary material for: Quantity and Variety of Fruit and Vegetable Intake with Changes in Measures of Adiposity among Community-Dwelling Chinese Older Adults
Source: Nutrients. 2023 Sep 22;15(19):4096. doi: 10.3390/nu15194096 (PMC10574446; doi:10.3390/nu15194096)
Supplement: Supplementary file 1 [file nutrients-15-04096-s001.zip › nutrients-2590373-supplementary.pdf]

Supplementary Materials

Table S1. Different kinds of fruits and vegetables [1-5].

Group	FFQ variables	Major bioactive components
**Vegetables**		
Cruciferous vegetables	Broccoli, Cabbage, Chinese kale, Bok choy, Cauliflower, Chinese mustard greens, Chinese radish, Chinese leaves	Vitamin C, Folate, Flavonoids, Glucosinolates
Green vegetables	Choy sum, Chinese spinach, Spinach, Watercress, Water spinach, Tong ho, Lettuce, Hairy melon, Winter melon, Bitter melon, Cucumber, Angled loofah, Celery, Green capsicum, Asparagus	Vitamin C, Folate, Flavonoids
Yellow/red vegetables	Tomato, Carrot, Pumpkin, Red capsicum	Carotene, Carotenoids
Allium vegetables	Onion, Chinese chives	Organosulfur
Beans and peas	Long bean, Snow peas, Mung bean sprout, Snap bean, Soybean sprout, Peas, Broad bean, Pea shoots	Isoflavones, Flavonoids, Peptides
Other vegetables	Mixed vegetable, Eggplant, White fungus, Chinese dried mushroom, Straw mushroom, Mushroom, Wood fungus, Black moss, Bamboo shoot	
**Fruits**		
Citrus fruits	Orange, Pomelo, Lemon, Grapefruit	Vitamin C, Carotenoids, Flavonoids
Berries and Drupes	Strawberry, Kiwi fruit, Grapes, Pomegranate, Plum, Cherry, Peach, Apricot, Longan, Dried red date, Dried prunes	Flavonoid, Anthocyanins
Pomes	Apple, Chinese pear, Persimmon, Papaya	Carotenoids, Flavonoid
Tropical fruits	Banana, Lychee, Mango, Pineapple	Vitamin C, Carotenoids
Melons	Watermelon, Honeydew Melons	

Table S2. Baseline characteristics of participants included and participants without follow-up information.

	Participants included (*n*=2,944)	Participants without follow-up information (*n*=754)	*p* [a]
Fruit intake, g/d	252.5±185.4	245.5±214.3	0.017
Vegetable intake. g/d	232.2±150.0	211.0±154.5	<0.001
Fruit variety	10±4	9±4	<0.001
Vegetable variety	24±6	22±6	<0.001
Age, y	71.8±4.8	74.5±5.7	<0.001
Male, %	52.0	54.1	0.301
Education, %			0.005
No education	18.3	22.4	
Primary or below	51.0	51.9	
Secondary or above	30.7	25.7	
Current smoker, %	6.4	9.2	0.008
Current alcohol drinker, %	14.0	12.5	0.276
Subjective social status [b], rung			
Community Ladder	6.9±2.2	6.6±2.4	0.027
Hong Kong Ladder	4.6±1.9	4.5±2.0	0.023
Number of chronic diseases, %			<0.001
0	62.1	53.0	
1	27.7	30.6	
≥2	10.3	15.4	
PASE score	95.7±44.0	83.7±40.6	<0.001
Energy intake, kcal/d	1866.7±577.1	1817.7±571.4	0.045
DQI-I	64.8±9.3	63.1±10.3	<0.001
Fiber intake, g/d	9.4±4.4	8.8±5.0	<0.001
Vitamin C, mg/d	159.4±96.9	152.6±118.1	<0.001
Weight, cm	58.9±9.6	58.0±10.3	0.021
BMI, kg/m^2	23.7±3.2	23.5±3.6	0.076
Waist circumference, cm	86.3±9.2	86.7±9.7	0.330
Fat mass, kg	17.2±5.2	16.9±5.5	0.195
Fat mass, %	29.3±7.2	29.2±7.4	0.644
Lean mass, kg	41.2±7.4	40.5±7.6	0.022
Lean mass, %	70.7±7.2	70.8±7.4	0.644

Abbreviation: BMI, body mass index; PASE, Physical Activity Scale for the Elderly; DQI-I, Diet quality index-International.

[a] *p* was assessed by *t* test or Mann-Whitney *U* test for continuous variables and Chi-Square for categorical variables.

[b] 10-rung self-anchoring scale (the lowest rung represents the most undesirable; the highest rung represents the most desirable state for their standing in the community/Hong Kong).

Table S3. Changes in measures of adiposity over 4 years in the Mr.OS and Ms.OS (Hong Kong) study.

	Baseline	The 4th year follow up	Change	p [a]
Weight, kg	58.9±9.6	58.2±9.8	-0.73±3.10	<0.001
BMI, kg/m^2	23.7±3.2	23.6±3.3	-0.15±1.24	<0.001
Waist circumference, cm	86.3±9.2	88.1±9.8	1.76±6.96	<0.001
Fat mass, kg	17.2±5.2	17.2±5.3	-0.01±2.13	0.726
Fat mass, %	29.3±7.1	29.6±7.3	0.29±2.58	<0.001
Lean mass, kg	41.2±7.4	40.5±7.4	-0.69±1.49	<0.001
Lean mass, %	70.7±7.2	70.4±7.3	-0.29±2.58	<0.001

[a] p was assessed by paired t test.

Table S4. Baseline characteristics of participants according to the quartiles of fruits and vegetables intake.

	Fruits intake [a]				*p*	Vegetables intake [a]				*p*
	**Q1**	**Q2**	**Q3**	**Q4**		**Q1**	**Q2**	**Q3**	**Q4**	
Fruits intake, g/day	93.2±38.9	182.9±20.4	262.7±31.8	470.3±238.5	<0.001	198.7±186.6	240.7±187.6	263.5±170.8	306.9±180.0	<0.001
Citrus fruits	31.9±28.6	85.1±52.3	111.6±60.4	187.9±168.1	<0.001	86.3±82.0	106.4±145.2	104.8±98.1	119.4±100.7	<0.001
Berries and Drupes	5.4±8.1	7.3±10.0	12.7±17.2	40.3±109.5	<0.001	11.6±54.2	17.9±70.1	16.9±56.1	19.3±47.2	<0.001
Pomes	27.2±23.9	49.3±41.5	71.5±49.0	116.0±86.6	<0.001	47.7±59.6	58.9±53.1	72.6±62.6	84.8±73.7	<0.001
Tropical fruits	21.5±20.7	31.0±30.9	51.1±44.5	92.3±157.2	<0.001	43.1±137.3	44.4±57.9	48.7±58.9	59.7±72.5	<0.001
Melons	7.3±10.2	10.3±16.1	15.9±22.8	33.9±81.9	<0.001	9.9±19.5	13.0±36.6	20.6±62.8	23.8±47.3	<0.001
Vegetables intake. g/day	187.8±140.6	215.0±131.5	247.0±156.4	278.7±154.8	<0.001	100.0±28.2	169.3±16.9	238.2±24.8	420.8±176.8	<0.001
Cruciferous vegetables	38.2±57.6	41.4±37.6	46.9±42.0	54.1±53.5	<0.001	18.1±12.7	32.8±18.6	45.5±24.4	84.1±77.3	<0.001
Green vegetables	105.4±96.0	118.6±88.4	134.9±103.5	146.8±89.1	<0.001	57.1±22.0	95.1±25.7	131.8±34.0	221.4±139.5	<0.001
Yellow/red vegetables	20.4±27.8	24.7±41.0	30.8±40.8	36.3±54.7	<0.001	11.7±9.8	18.8±14.2	27.6±22.1	54.1±73.7	<0.001
Allium vegetables	2.9±5.0	3.6±5.0	5.5±10.7	6.3±10.4	<0.001	1.7±2.7	3.0±3.9	4.9±5.7	8.6±14.0	<0.001
Beans and peas	12.8±23.3	15.6±21.8	17.8±18.7	22.5±23.5	<0.001	6.7±5.8	11.7±13.6	17.6±13.6	32.7±35.7	<0.001
Other vegetables	9.0±12.4	12.1±34.6	12.7±15.4	15.0±21.4	<0.001	5.1±5.1	8.6±8.2	12.1±9.6	22.9±41.2	<0.001
Fruits variety	9±4	10±4	11±4	12±4	<0.001	9±4	10±4	11±4	11±4	<0.001
Vegetables variety	23±6	24±6	25±6	25±6	<0.001	22±6	24±6	25±6	27±6	<0.001
Age, year	71.9±4.8	71.7±4.9	71.8±4.7	71.7±4.9	0.703	72.1±5.0	72.0±4.8	71.5±4.6	71.6±4.8	0.088
Male, %	51.8	52.2	52.0	52.0	0.999	52.0	52.0	52.0	52.0	1.000
Education, %					<0.001					0.015
No education	20.9	18.8	17.3	16.3		21.2	18.3	16.2	17.5	
Primary or below	53.2	53.0	51.0	46.7		51.4	53.1	51.4	47.9	
Secondary or above	25.9	28.2	31.8	37.0		27.4	28.5	32.5	34.6	
Current smoker, %	10.8	7.6	3.7	3.7	0.023	8.6	6.8	5.6	4.6	0.013
Current alcohol drinker, %	14.7	13.4	15.1	12.8	0.531	12.5	13.2	16.2	14.1	0.200
Subjective social status [b], rung										
Community Ladder	6.7±2.2	6.8±2.2	7.0±2.1	6.9±2.2	0.057	6.8±2.1	6.9±2.1	6.9±2.2	6.9±2.3	0.950
Hong Kong Ladder	6.9±2.2	6.9±2.2	6.8±2.1	6.9±2.2	0.592	4.5±1.8	4.6±1.8	4.6±1.8	4.6±1.9	0.747
Number of chronic diseases, %					0.762					0.635
0	61.3	62.2	61.0	63.8		62.0	63.0	61.1	62.0	
1	27.2	28.3	28.8	26.5		27.8	28.5	27.5	27.0	
≥2	11.6	9.5	10.2	9.8		10.2	8.4	11.4	11.0	
PASE score	91.3±44.8	93.8±42.2	94.4±43.5	99.4±45.2	0.002	88.4±41.8	90.3±39.3	97.2±46.2	103.1±46.8	<0.001
Energy intake, kcal/day	1673±533	1769±520	1898±552	2123±589	<0.001	1606±497	1818±541	1916±545	2125±597	<0.001
DQI-I	58.9±9.4	64.0±8.0	66.4±8.1	70.0±7.9	<0.001	58.5±9.3	64.5±8.1	66.5±7.8	69.8±8.0	<0.001
Fiber intake, g/day	6.5±4.4	8.3±3.8	9.8±3.6	13.1±5.2	<0.001	6.2±3.8	8.3±4.1	9.7±3.3	13.5±5.2	<0.001
Vitamin C, mg/day	99.1±70.2	136.7±72.0	167.5±67.5	233.8±114.5	<0.001	103.6±79.2	142.0±95.7	162.7±64.2	229.1±98.5	<0.001

	Fruits intake [a]				*p*	Vegetables intake [a]				*p*
	**Q1**	**Q2**	**Q3**	**Q4**		**Q1**	**Q2**	**Q3**	**Q4**	
Weight, kg	58.1±9.8	58.7±8.9	59.2±9.8	59.6±9.9	0.043	58.3±9.6	58.4±9.1	59.5±9.5	59.3±10.2	0.032
BMI, kg/m^2	23.5±3.3	23.8±3.1	23.8±3.2	23.8±3.2	0.130	23.6±3.2	23.5±3.1	23.9±3.1	23.9±3.3	0.161
Waist circumference, cm	85.9±0.6	86.6±8.8	86.5±9.1	86.4±9.1	0.503	86.3±9.3	86.2±8.8	86.2±8.9	86.6±9.6	0.776
Fat mass, kg	16.9±5.3	17.2±5.0	17.4±5.3	17.2±5.2	0.208	17.1±5.1	17.0±5.3	17.3±5.1	17.3±5.3	0.595
Fat mass, %	29.1±7.3	29.5±7.3	29.5±7.1	29.1±7.0	0.480	29.4±7.2	29.2±7.4	29.3±7.1	29.2±7.0	0.963
Lean mass, kg	40.7±7.4	41.0±7.2	41.3±7.4	41.8±7.7	0.047	40.7±7.5	40.9±7.1	41.7±7.5	41.5±7.7	0.040
Lean mass, %	70.9±7.3	70.5±7.3	70.5±7.0	70.9±7.0	0.480	70.6±7.2	70.8±7.4	70.7±7.1	70.8±7.0	0.963

Abbreviation: PASE, Physical Activity Scale for the Elderly; DQI-I, DQI-I, Diet quality index-International; BMI, body mass index.

[a] Fruits and vegetables intake: sex-specific quartile. [b] 10-rung self-anchoring scale (the lowest rung represents the most undesirable; the highest rung represents the most desirable state for their standing in the community/Hong Kong).

Table S5. Baseline characteristics of participants according to the quartiles of fruits and vegetables variety.

	Fruits variety [a]				*p*	Vegetables variety [a]				*p*
	Q1	Q2	Q3	Q4		Q1	Q2	Q3	Q4	
Fruits intake, g/day	190.7±138.6	241.4±185.7	279.1±217.5	311.5±168.3	<0.001	227.2±161.8	230.6±169.3	265.1±224.2	292.2±166.5	<0.001
Citrus fruits	97.2±101.4	105.5±141.8	105.4±90.7	109.5±85.5	<0.001	107.2±104.2	96.6±104.0	105.8±133.0	106.6±86.7	<0.001
Berries and Drupes	4.9±13.5	11.8±45.5	22.0±78.7	30.2±71.0	<0.001	10.6±43.2	14.4±46.3	17.2±63.5	25.1±73.3	<0.001
Pomes	45.4±56.4	63.9±63.0	72.2±62.7	86.6±68.9	<0.001	55.1±60.9	61.3±63.3	67.9±57.5	82.6±73.4	<0.001
Tropical fruits	34.2±53.9	45.4±60.3	57.4±63.5	61.9±61.2	<0.001	43.5±70.7	44.2±51.0	53.8±57.1	54.9±51.3	<0.001
Melons	8.9±32.5	14.7±39.3	22.1±64.1	23.3±33.9	<0.001	10.8±32.3	14.1±28.7	20.4±63.2	23.0±43.0	<0.001
Vegetables intake. g/day	207.1±146.9	233.2±162.8	236.5±136.9	256.2±145.9	<0.001	187.9±144.6	224.5±151.6	244.6±145.0	280.9±144.5	<0.001
Cruciferous vegetables	37.6±48.6	47.3±54.0	46.8±42.4	49.3±47.1	<0.001	30.7±33.2	43.7±45.6	51.7±58.5	56.8±50.0	<0.001
Green vegetables	120.6±101.5	128.1±102.2	125.3±73.3	132.6±101.9	<0.001	115.4±113.9	124.8±97.6	128.0±82.8	140.1±80.9	<0.001
Yellow/red vegetables	24.2±42.7	28.6±51.7	28.1±37.1	32.0±33.0	<0.001	22.6±43.4	26.5±45.4	30.0±44.8	34.2±33.7	<0.001
Allium vegetables	3.0±5.9	4.1±8.9	5.4±10.5	6.1±6.9	<0.001	2.7±7.1	3.9±8.7	4.7±7.6	7.6±9.5	<0.001
Beans and peas	13.1±21.2	15.6±23.8	19.5±21.8	21.7±20.3	<0.001	9.5±17.1	15.6±28.6	18.4±15.4	27.1±23.4	<0.001
Other vegetables	9.3±18.4	10.8±13.9	13.2±32.1	16.5±23.2	<0.001	7.7±12.8	11.0±13.9	13.1±29.8	18.1±27.8	<0.001
Fruits variety	5±2	9±1	12±1	16±2	<0.001	7±3	9±3	11±3	14±3	<0.001
Vegetables variety	20±6	23±5	26±5	30±5	<0.001	16±3	23±1	27±1	33±3	<0.001
Age, year	72.5±5.0	72.3±5.0	71.4±4.6	70.8±4.3	<0.001	72.9±4.9	71.9±5.1	71.4±4.6	70.8±4.3	<0.001
Male, %	43.0	52.3	53.4	61.0	<0.001	43.8	49.7	55.6	60.2	<0.001
Education, %					<0.001					<0.001
No education	26.6	18.3	16.9	10.0		25.3	18.4	16.0	12.3	
Primary or below	51.9	53.2	50.1	47.7		50.4	55.3	49.0	49.5	
Secondary or above	21.5	28.5	33.1	42.3		24.3	26.3	35.0	38.2	
Current smoker, %	6.2	6.8	6.3	6.2	0.963	5.2	7.3	6.7	6.3	0.352
Current alcohol drinker, %	9.3	14.6	13.6	19.4	<0.001	10.4	11.4	15.1	20.0	<0.001
Subjective social status [b], rung										
Community Ladder	6.9±2.3	6.8±2.2	6.9±2.1	6.9±2.1	0.493	6.9±2.2	6.9±2.2	6.8±2.1	6.9±2.2	0.592
Hong Kong Ladder	4.5±2.0	4.5±1.8	4.6±1.8	4.7±1.8	0.004	4.4±1.9	4.5±2.0	4.7±1.8	4.8±1.7	<0.001
Number of chronic diseases, %					0.161					0.449
0	58.5	62.3	64.2	63.6		62.5	59.1	64.7	61.3	
1	30.4	27.6	24.8	27.9		27.3	30.4	25.3	28.3	
≥2	11.2	10.1	11.0	8.5		10.3	10.5	10.0	10.4	
PASE score	89.9±43.8	91.7±40.7	97.4±46.5	101.8±44.8	<0.001	90.2±41.9	91.7±42.2	97.5±46.8	100.3±44.2	<0.001
Energy intake, kcal/day	1692±519	1816±547	1878±539	2134±629	<0.001	1675±529	1784±526	1930±549	2118±617	<0.001
DQI-I	62.7±10.0	64.6±8.8	66.3±8.7	66.1±9.2	<0.001	63.0±9.6	64.2±9.2	65.6±8.9	66.8±8.9	<0.001
Fiber intake, g/day	7.8±4.1	9.0±4.4	10.0±5.7	11.3±4.8	<0.001	7.8±4.0	8.7±4.3	9.9±4.8	11.8±5.7	<0.001
Vitamin C, mg/day	136.9±79.4	158.4±101.0	166.1±100.2	180.0±100.7	<0.001	139.7±78.5	150.2±85.8	165.9±106.1	185.9±109.4	<0.001

	Fruits variety [a]				*p*	Vegetables variety [a]				*p*
	**Q1**	**Q2**	**Q3**	**Q4**		**Q1**	**Q2**	**Q3**	**Q4**	
Weight, kg	58.2±9.7	58.4±9.4	59.3±9.6	59.9±9.7	0.004	58.2±9.6	58.8±10.0	59.1±9.4	59.7±9.4	0.024
BMI, kg/m^2	23.8±3.4	23.6±3.2	23.8±3.0	23.7±3.1	0.875	23.8±3.3	23.8±3.4	23.7±3.0	23.7±3.1	0.926
Waist circumference, cm	86.5±9.1	86.3±9.3	86.4±9.1	86.0±9.2	0.807	86.7±9.4	86.8±9.4	85.9±9.0	85.9±8.9	0.102
Fat mass, kg	17.6±5.6	16.9±5.2	17.4±5.0	16.8±4.9	0.007	17.5±5.5	17.3±5.3	17.0±4.9	16.9±5.1	0.133
Fat mass, %	30.3±7.5	29.1±7.2	29.6±7.0	28.2±6.7	<0.001	30.1±7.4	29.4±7.1	29.0±6.9	28.6±7.1	<0.001
Lean mass, kg	40.2±7.4	41.0±7.1	41.4±7.7	42.5±7.5	<0.001	40.2±7.2	41.1±7.5	41.6±7.6	42.1±7.4	<0.001
Lean mass, %	69.7±7.4	70.9±7.2	70.4±7.0	71.8±6.7	<0.001	69.9±7.4	70.6±7.1	71.0±6.9	71.4±7.0	<0.001

Abbreviation: PASE, Physical Activity Scale for the Elderly; DQI-I, DQI-I, Diet quality index-International; BMI, body mass index. [a] Fruits variety score: Q1 for 0-6 specific types of fruits that consumed; Q2 for 7-10 specific types; Q3 for 11-13 specific types; Q4 for more than 14 specific types; vegetable variety score: Q1 for 0-20 specific types of vegetables that consumed; Q2 for 21-24 specific types; Q3 for 25-29 specific types; Q4 for more than 30 specific types. [b] 10-rung self-anchoring scale (the lowest rung represents the most undesirable and the highest rung represents the most desirable state for their standing in the community/Hong Kong).

Table S6. Spearman's rank correlation coefficient between the intake and variety of fruits and vegetables and intakes of other food groups.

	Fruit intake		Vegetable intake		Fruit variety		Vegetable variety	
	rho	*p*	rho	*p*	rho	*p*	rho	*p*
Red meat	0.0996	<0.001	0.1579	<0.001	0.1773	<0.001	0.2102	<0.001
Processed meat	0.1361	<0.001	0.1889	<0.001	0.1257	<0.001	0.1646	<0.001
Poultry	0.1699	<0.001	0.1930	<0.001	0.1846	<0.001	0.2052	<0.001
Freshwater fish	0.1772	<0.001	0.2775	<0.001	0.1206	<0.001	0.1434	<0.001
Seafood	0.1612	<0.001	0.2497	<0.001	0.1597	<0.001	0.1309	<0.001
Egg	0.1217	<0.001	0.2134	<0.001	0.1211	<0.001	0.1206	<0.001
Milk	0.1954	<0.001	0.1959	<0.001	0.1596	<0.001	0.1702	<0.001
Whole grains	0.1088	<0.001	0.1355	<0.001	0.0379	0.040	0.0672	<0.001
Refined grains	0.0338	0.067	0.1051	<0.001	0.0249	0.177	0.0777	<0.001
Oil	0.0593	0.001	0.0475	0.010	0.1002	<0.001	0.1459	<0.001
Condiments	0.0993	<0.001	0.0747	<0.001	0.2262	<0.001	0.1815	<0.001

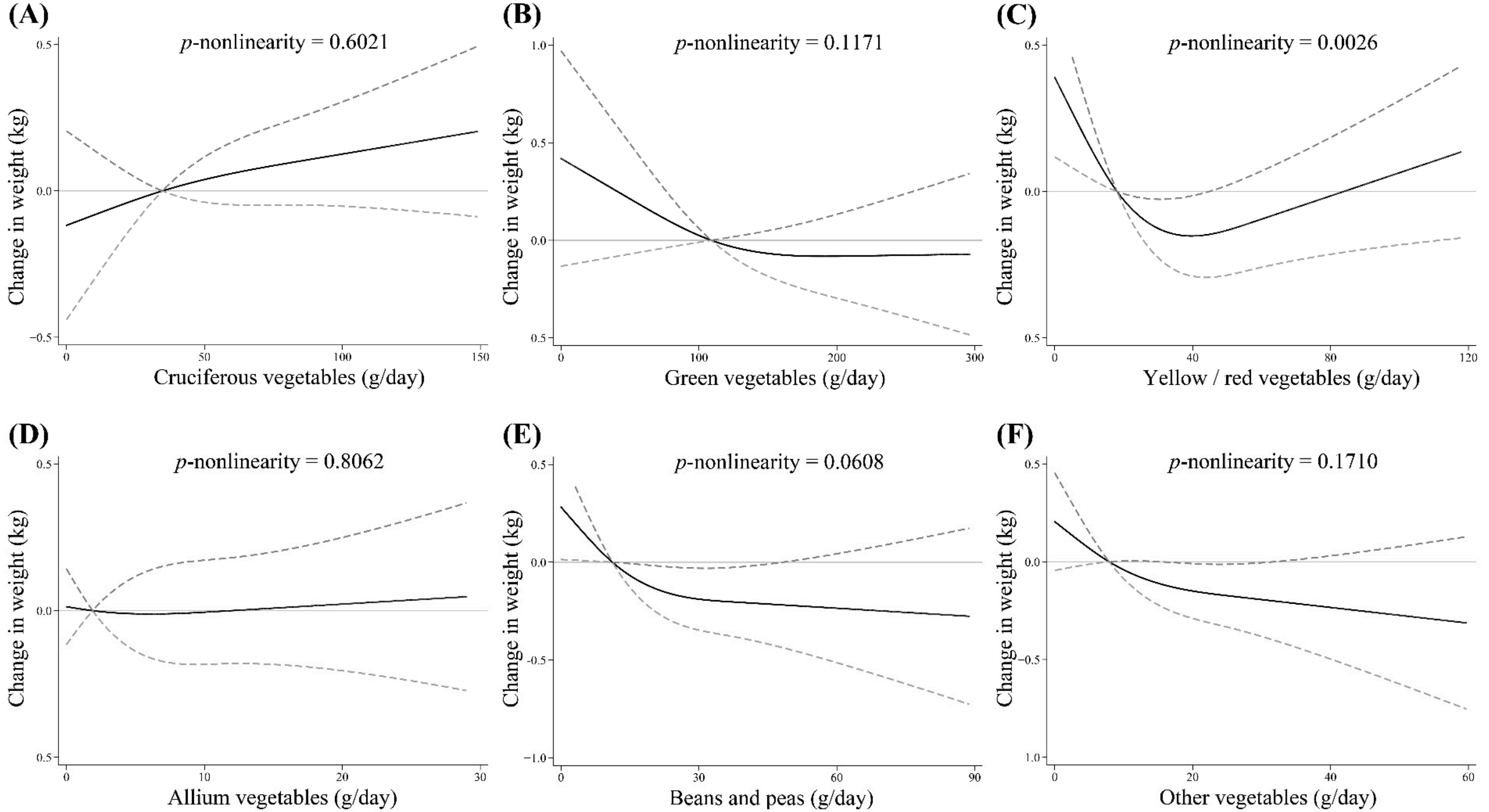

Figure S1. Associations of various kinds of vegetables intake with 4-year change in body weight.

(A) for cruciferous vegetables; (B) for green vegetables; (C) for yellow/red vegetables; (D) for allium vegetables; (E) for beans and peas; (F) for other vegetables. Vegetable intake was coded using restricted cubic spline functions with three knots located at the 10th, 50th, and 90th percentiles and the median was used as reference. Adjusted for age, sex, education level, smoking status, alcohol drinking, subjective social status (community ladder and Hong Kong ladder), physical activity and number of chronic diseases, energy intake, total vegetable intake, DQI-I, baseline body weight.

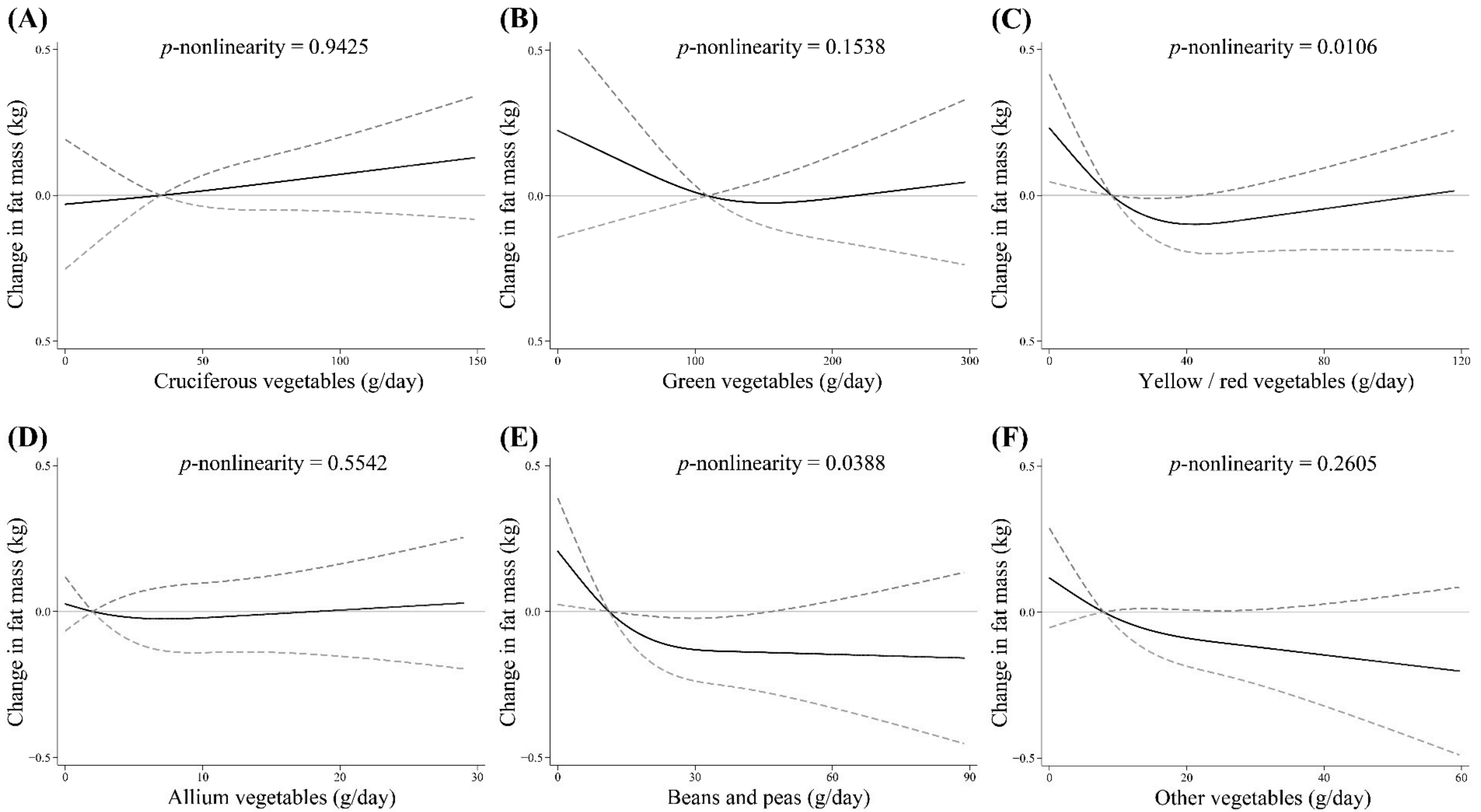

Figure S2. Associations of various kinds of vegetables intake with 4-year change in fat mass.

(A) for cruciferous vegetables; (B) for green vegetables; (C) for yellow/red vegetables; (D) for allium vegetables; (E) for beans and peas; (F) for other vegetables. Vegetable intake was coded using restricted cubic spline functions with three knots located at the 10th, 50th, and 90th percentiles and the median was used as reference. Adjusted for age, sex, education level, smoking status, alcohol drinking, subjective social status (community ladder and Hong Kong ladder), physical activity and number of chronic diseases, energy intake, total vegetable intake, DQI-I, baseline fat mass.

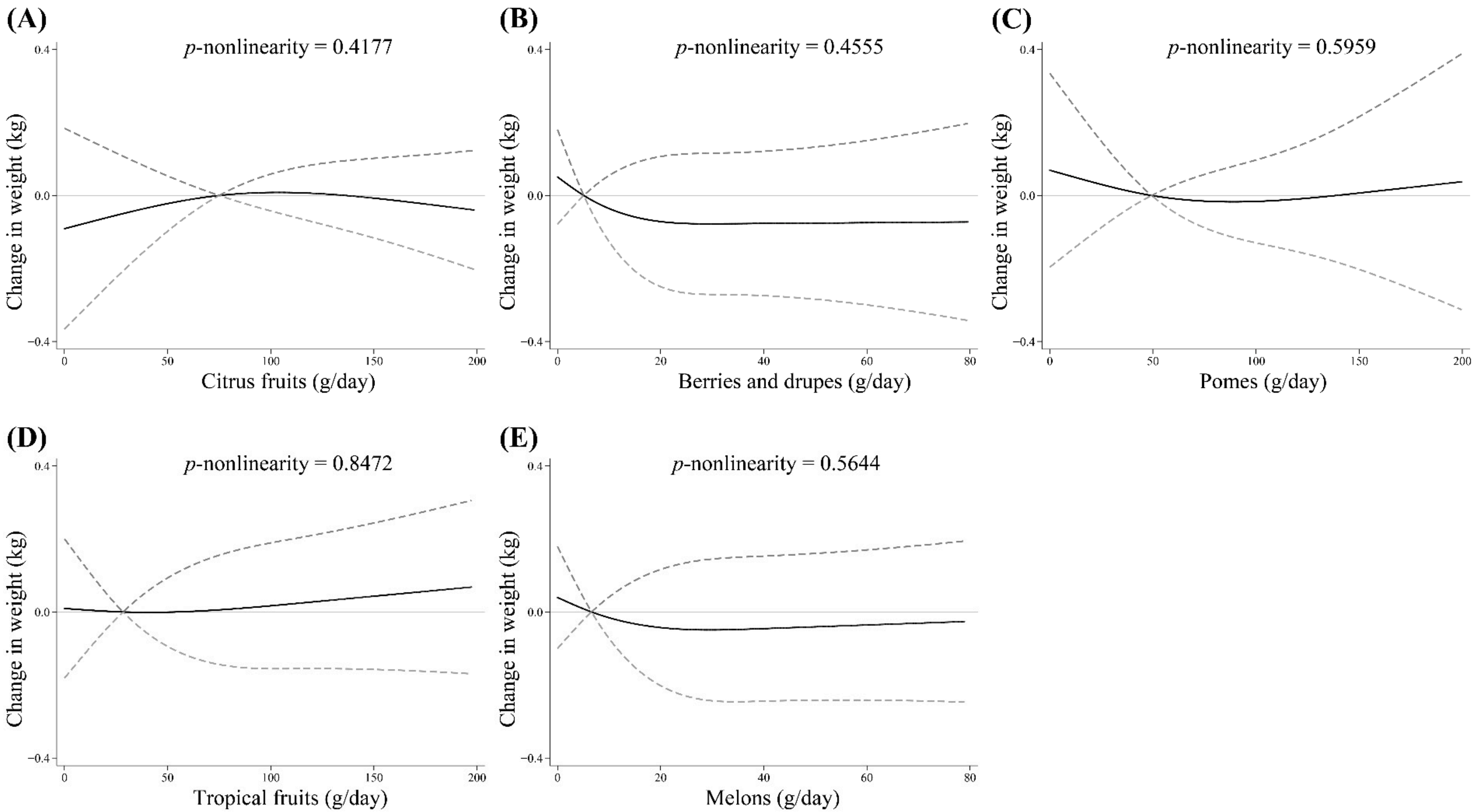

Figure S3. Associations of various kinds of fruits intake with 4-year change in body weight.

(A) for citrus fruits; (B) for berries and drupes; (C) for pomes; (D) for tropical fruits; (E) for melons. Fruit intake was coded using restricted cubic spline functions with three knots located at the 10th, 50th, and 90th percentiles and the median was used as reference. Adjusted for age, sex, education level, smoking status, alcohol drinking, subjective social status (community ladder and Hong Kong ladder), physical activity and number of chronic diseases, energy intake, total fruit intake, DQI-I, baseline body weight.

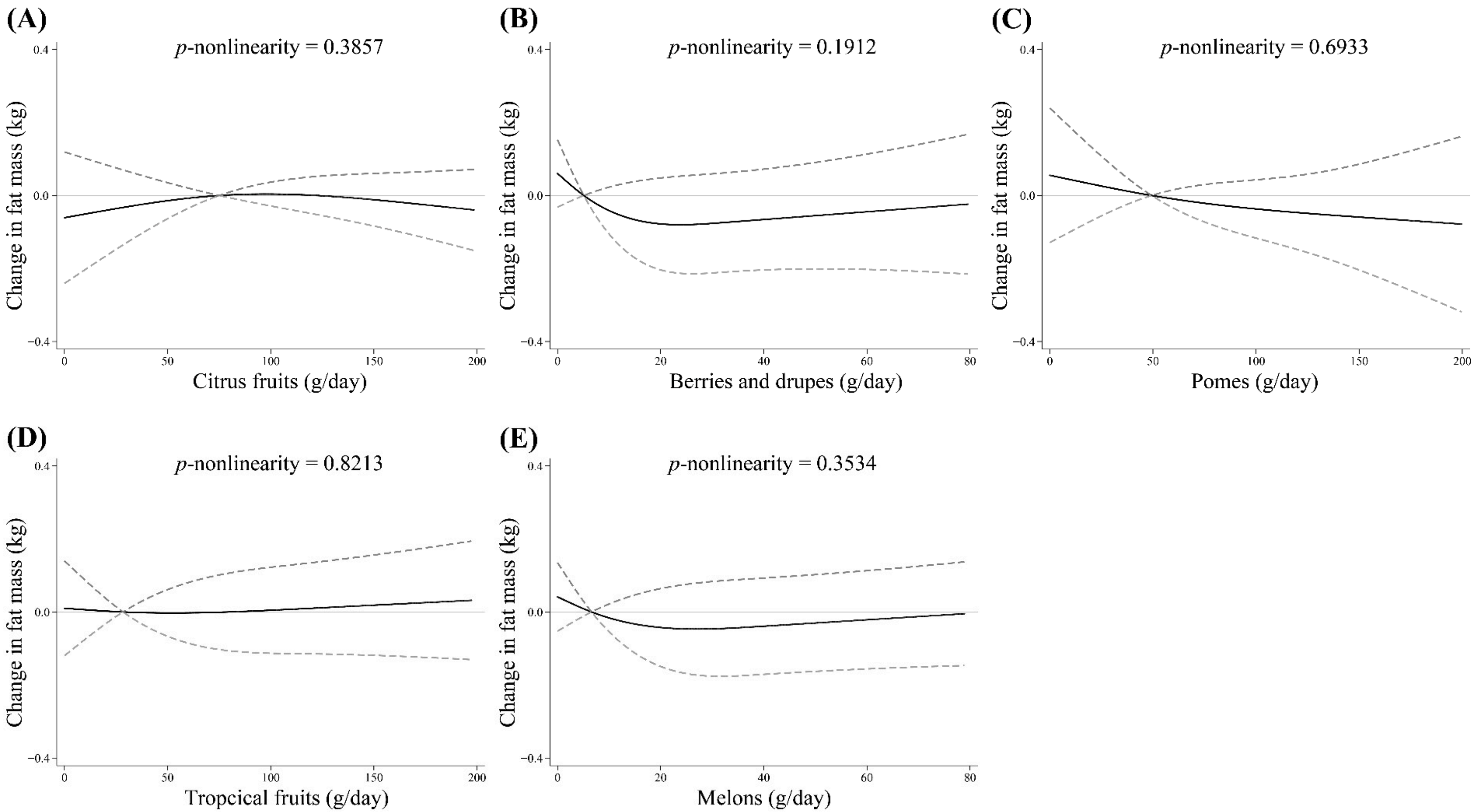

Figure S4. Associations of various kinds of fruits intake with 4-year change in fat mass.

(A) for citrus fruits; (B) for berries and drupes; (C) for pomes; (D) for tropical fruits; (E) for melons. Fruit intake was coded using restricted cubic spline functions with three knots located at the 10th, 50th, and 90th percentiles and the median was used as reference. Adjusted for age, sex, education level, smoking status, alcohol drinking, subjective social status (community ladder and Hong Kong ladder), physical activity and number of chronic diseases, energy intake, total fruit intake, DQI-I, baseline fat mass.

Table S7. Coefficient of association between the intake and variety of fruits and vegetables with changes in measures of adiposity over 4 years. [a]

	Fruit intake, per 100 g/day			Vegetable intake, per 100 g/day			Fruit variety			Vegetable variety		
	β	*SE*	*p*	*β*	*SE*	*p*	*β*	*SE*	*p*	*β*	*SE*	*p*
Weight, kg	-0.032	0.035	0.356	0.025	0.042	0.559	0.001	0.015	0.954	-0.001	0.009	0.985
BMI, kg/m^2	-0.012	0.014	0.379	0.010	0.017	0.560	0.001	0.006	0.955	-0.002	0.004	0.617
Waist circumference, cm	-0.059	0.073	0.423	0.008	0.090	0.930	-0.014	0.032	0.664	0.016	0.020	0.425
Fat mass, kg	-0.003	0.024	0.908	0.016	0.029	0.582	-0.004	0.010	0.717	-0.005	0.007	0.465
Fat mass, %	0.004	0.029	0.899	0.015	0.035	0.669	0.001	0.013	0.947	-0.003	0.008	0.663
Lean mass, kg	-0.006	0.016	0.715	-0.006	0.020	0.765	-0.001	0.007	0.905	0.002	0.005	0.663
Lean mass, %	-0.004	0.029	0.899	-0.015	0.035	0.669	-0.001	0.013	0.947	0.003	0.008	0.663

[a] The multivariate linear association models were adjusted for age, sex, education level, smoking status, alcohol drinking, subjective social status (community ladder and Hong Kong ladder), physical activity and number of chronic diseases, energy intake, DQI-I, baseline measure of adiposity; additionally adjusted for fruit and vegetable variety for their intake or adjusted fruit and vegetable intake for their variety.

Table S8. Associations between the intake and variety of fruits and vegetables with weight loss.

	***n* (%)**	**Weight loss [a], OR (95%CI)**		
		**Model 1**	**Model 2**	**Model 3**
Fruit intake				
Q1	149 (20.3)	1.00 (Ref)	1.00 (Ref)	1.00 (Ref)
Q2	149 (20.2)	1.00 (0.77, 1.30)	1.04 (0.80, 1.36)	1.04 (0.79, 1.36)
Q3	160 (21.7)	1.09 (0.85, 1.41)	1.15 (0.87, 1.51)	1.15 (0.87, 1.52)
Q4	128 (17.4)	0.82 (0.63, 1.07)	0.88 (0.65, 1.20)	0.90 (0.66, 1.22)
p-trend		0.149	0.407	0.468
Vegetable intake				
Q1	153 (20.8)	1.00 (Ref)	1.00 (Ref)	1.00 (Ref)
Q2	151 (20.5)	0.97 (0.75, 1.25)	1.03 (0.79, 1.35)	1.03 (0.79, 1.36)
Q3	137 (18.6)	0.85 (0.65, 1.10)	0.90 (0.68, 1.19)	0.90 (0.67, 1.20)
Q4	145 (19.7)	0.95 (0.73, 1.23)	1.06 (0.78, 1.43)	1.05 (0.77, 1.44)
p-trend		0.644	0.784	0.803
Fruit variety				
Q1	153 (20.6)	1.00 (Ref)	1.00 (Ref)	1.00 (Ref)
Q2	176 (20.5)	1.02 (0.80, 1.31)	1.03 (0.80, 1.32)	1.01 (0.79, 1.31)
Q3	153 (21.0)	1.10 (0.85, 1.43)	1.12 (0.86, 1.46)	1.11 (0.85, 1.45)
Q4	104 (17.0)	0.86 (0.64, 1.14)	0.86 (0.64, 1.16)	0.86 (0.63, 1.16)
p-trend		0.470	0.567	0.536
Vegetable variety				
Q1	169 (20.9)	1.00 (Ref)	1.00 (Ref)	1.00 (Ref)
Q2	154 (22.5)	1.15 (0.89, 1.48)	1.17 (0.91, 1.51)	1.17 (0.91, 1.52)
Q3	138 (17.0)	0.86 (0.66, 1.11)	0.88 (0.68, 1.14)	0.88 (0.68, 1.15)
Q4	125 (19.7)	1.05 (0.81, 1.38)	1.10 (0.83, 1.45)	1.10 (0.83, 1.46)
p-trend		0.804	0.953	0.951

Abbreviation: OR, odd ratio; 95%CI, 95% confidence interval; Ref, reference.

[a] Weight loss was defined as a 5% or greater loss of body weight.

[b] Model 1: adjusted for age and sex.

[c] Model 2: adjusted for Model 1 + education level, smoking status, alcohol drinking, subjective social status (community ladder and Hong Kong ladder), physical activity and number of chronic diseases, energy intake, DQI-I and baseline body weight.

[d] Model 3: adjusted for Model 2 + fruit and vegetable variety for their intake or adjusted fruit and vegetable intake for their variety.

Table S9. Sensitivity analysis of the association between the intake and variety of fruits and vegetables with weight gain over 4 years.

	Weight gain [a], OR (95%CI)			
	**Model 3 [d]**	**Model 3a [c]**	**Model 3b [d]**	**Model 3c [e]**
Fruit intake				
Q1	1.00 (Ref)	1.00 (Ref)	1.00 (Ref)	1.00 (Ref)
Q2	1.06 (0.73, 1.54)	1.07 (0.74, 1.55)	1.02 (0.70, 1.47)	1.05 (0.73, 1.52)
Q3	1.00 (0.67, 1.48)	0.99 (0.67, 1.47)	0.92 (0.62, 1.37)	0.94 (0.64, 1.40)
Q4	0.93 (0.61, 1.42)	0.97 (0.64, 1.49)	0.90 (0.59, 1.38)	0.95 (0.62, 1.45)
Vegetable intake				
Q1	1.00 (Ref)	1.00 (Ref)	1.00 (Ref)	1.00 (Ref)
Q2	0.81 (0.56, 1.17)	0.82 (0.57, 1.18)	0.78 (0.54, 1.12)	0.80 (0.55, 1.16)
Q3	**0.55 (0.36, 0.83)**	**0.57 (0.38, 0.87)**	**0.53 (0.35, 0.80)**	**0.56 (0.37, 0.84)**
Q4	0.88 (0.58, 1.33)	0.89 (0.59, 1.34)	0.81 (0.53, 1.22)	0.84 (0.56, 1.28)
Fruit variety				
Q1	1.00 (Ref)	1.00 (Ref)	1.00 (Ref)	1.00 (Ref)
Q2	1.04 (0.74, 1.48)	1.05 (0.74, 1.49)	1.03 (0.73, 1.46)	1.05 (0.75, 1.49)
Q3	1.04 (0.71, 1.50)	1.02 (0.71, 1.48)	1.01 (0.70, 1.47)	1.04 (0.72, 1.50)
Q4	0.75 (0.49, 1.15)	0.78 (0.51, 1.19)	0.77 (0.50, 1.18)	0.79 (0.52, 1.20)
Vegetable variety				
Q1	1.00 (Ref)	1.00 (Ref)	1.00 (Ref)	1.00 (Ref)
Q2	0.90 (0.61, 1.32)	0.87 (0.60, 1.28)	0.88 (0.54, 1.12)	0.86 (0.59, 1.26)
Q3	1.19 (0.84, 1.70)	1.23 (0.87, 1.75)	0.53 (0.35, 0.80)	1.25 (0.88, 1.77)
Q4	1.04 (0.69, 1.54)	1.03 (0.69, 1.53)	0.81 (0.53, 1.22)	1.03 (0.69, 1.53)

Abbreviation: OR, odd ratio; 95%CI, 95% confidence interval; Ref, reference.

[a] Weight gain" was defined as a 5% or greater gain of body weight.

[b] Model 3: adjusted for age and sex, education level, smoking status, alcohol drinking, subjective social status (community ladder and Hong Kong ladder), physical activity and number of chronic diseases, energy intake, DQI-I and baseline body weight, additionally adjusted for fruit and vegetable variety for their intake or adjusted for fruit and vegetable intake for their variety.

[c] Model 3a: additionally adjusted for cooking oil and condiment intake.

[d] Model 3b: additionally adjusted for red and processed meat intake.

[e] Model 3c: exclude those not within the mean ±3SD of fruit and vegetable intake (*n*=22).

Reference

1. Slavin, J.L.; Lloyd, B. Health benefits of fruits and vegetables. *Adv Nutr* **2012**, *3*, 506-516, https://doi.org/10.3945/an.112.002154.
2. González-Castejón, M.; Rodriguez-Casado, A. Dietary phytochemicals and their potential effects on obesity: a review. *Phamacol Res* **2011**, *64*, 438-455. https://doi.org/10.1016/j.phrs.2011.07.004.
3. Eichholzer M, Lüthy J, Gutzwiller F, Stähelin HB. The role of folate, antioxidant vitamins and other constituents in fruit and vegetables in the prevention of cardiovascular disease: the epidemiological evidence. *Int J Vitam Nutr Res* **2001**,*71*:5-17. https://doi.org/10.1024/0300-9831.71.1.5
4. Zhu, F.; Du, B.; Xu, B. Anti-inflammatory effects of phytochemicals from fruits, vegetables, and food legumes: A review. *Crit Rev Food Sci Nutr* **2018**, *58*, 1260-1270, https://doi.org/10.1080/10408398.2016.1251390.
5. Lai, H.T.; Threapleton, D.E.; Day, A.J.; Williamson, G.; Cade, J.E.; Burley, V.J. Fruit intake and cardiovascular disease mortality in the UK Women's Cohort Study. *Eur J Epidemiol* **2015**, *30*, 1035-1048, https://doi.org/10.1007/s10654-015-0050-5.
